# Monitoring deep inspiration breath hold for left‐sided localized breast cancer radiotherapy with an in‐house developed laser distance meter system

**DOI:** 10.1002/acm2.12137

**Published:** 2017-07-29

**Authors:** Christer A. Jensen, Tatiana Abramova, Jomar Frengen, Jo‐Åsmund Lund

**Affiliations:** ^1^ Department of Oncology Ålesund Hospital Ålesund Norway; ^2^ Clinic of Oncology St. Olavs Hospital Trondheim University Hospital Trondheim Norway; ^3^ Department of Cancer Research and Molecular Medicine Norwegian University of Science and Technology Trondheim Norway

**Keywords:** breast cancer radiation therapy, DIBH, laser distance measurer, respiratory gating

## Abstract

Deep inspiration breath hold (DIBH) in left‐sided breast cancer radiotherapy is a technique to reduce cardiac and pulmonary doses while maintaining target coverage. This study aims at evaluating an in‐house developed DIBH system. Free‐breathing (FB) and DIBH plans were generated for 22 left‐sided localized breast cancer patients who had radiation therapy (RT) after breast‐conserving surgery. All patients were treated utilizing an in‐house laser distance measuring system. 50 Gy was prescribed, and parameters of interest were target coverage, left anterior descending coronary artery, (LAD) and heart doses. Portal images were acquired and the reproducibility and stability of DIBH treatment were compared to FB. The comparing result shows there is a significant reduction in all LAD and heart dose statistics for DIBH compared to FB plans without compromising the target coverage. The maximum LAD dose was reduced from 43.7 Gy to 29.0 Gy and the volume of the heart receiving >25 Gy was reduced from 3.3% to 1.0% using the in‐house system, both statistically significant. The in‐house system gave a reproducible and stable DIBH treatment where the systematic error ∑, and random error *σ*, were less than 2.2 mm in all directions, but were not significantly better than at FB. The system was well tolerated and all patients completed their treatment sessions with DIBH.

## 1 INTRODUCTION

Clinical importance of radiation‐induced heart disease is well known, and there is growing evidence of a relation between radiotherapy (RT) and cardiovascular events.^1^, ^2^, ^3^, ^4^ Increased morbidity and mortality rates from cardiovascular damage may lower the survival rates.^5^ RT for left‐sided breast cancer may deliver a dose to the heart and lung. Excluding the heart from the field might compromise the dose to the target, but by means of the deep inspiration breath hold (DIBH) technique it is possible to reduce the cardiopulmonary doses while maintaining the prescribed dose to the breast.^6^, ^7^ The method is well established and several groups have previously reported beneficial results using DIBH.^6^, ^8^, ^9^, ^10^, ^11^, ^12^, ^13^, ^14^ Since DIBH is capable of decreasing dose to the heart; it is also assumed that the long‐term risk of developing cardiac damage is reduced.

The pathogenesis of radiation‐induced cardiovascular damage from animal studies have shown microvascular disease causing chronic ischemic heart disease, and macrovascular disease causing development of age‐related atherosclerosis in the coronary arteries.^2^ There is also new evidence of high‐grade coronary artery stenosis in mid and distal left anterior descending artery (LAD) in hotspot areas for radiation, and a four‐ to seven‐fold increase has been shown.^15^ A recent study has found increased use of percutaneous coronary intervention in patients treated with modern radiotherapy techniques, but this risk was limited to women with previous cardiac disease.^1^, ^16^ With free‐breathing (FB) radiotherapy parts of LAD might receive up to 50 Gy, and even with DIBH there can be a very high dose given to parts of the LAD for some of the patients. It is still unknown whether it is the mean dose to the heart, the high doses to the coronary arteries or the combination of both which causes an increased number of deaths from cardiac disease in left‐sided breast cancer that have undergone RT.

There are several commercial systems that offer the possibility to perform DIBH. The Active Breathing Coordinator system (Elekta, Crawley, UK) uses a spirometer where the patient makes use of a mouthpiece that closes a valve to ensure a standardized air‐volume into the patient's lungs. However, the spirometer technique has been reported to be less comfortable by the patients and Nissen et al. reported that 22 of 166 patients could not tolerate this system mainly due to the mouthpiece or to psychological reasons.^9^, ^17^, ^18^ The Real‐Time Positioning Management system (Varian, Palo Alto, USA) is less invasive, and relies on a box with infrared markers that is placed on the patients xiphoid process. The position of the box is tattooed on the patient, since its placement can influence the breath hold and could increase the dose to the skin if placed within the field borders due to the build‐up effect. Noninvasive systems like the Catalyst (C‐RAD Positioning, Uppsala, Sweden) and GateRT (Vision RT Ltd, London, UK) have recently entered the market.^19^, ^20^, ^21^ These systems project a light‐pattern onto the patient which is scanned by one or two CCD cameras. A high‐resolution 3D model of the patient can be reconstructed and used to perform gating when using these kinds of systems. The UK HeartSpare study relies on equipment‐free voluntary breath hold using skin surface marks as fiducials, and the technique has been shown to be effective and reproducible.^22^, ^23^, ^24^

**Figure 1:**
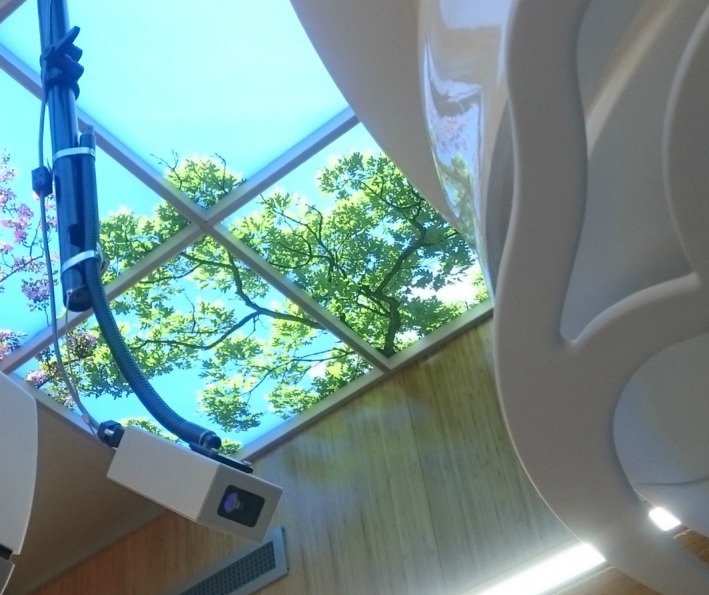
Laser measurer mounted on the ceiling in the treatment room.

We have previously published data on an in‐house developed noninvasive DIBH system based upon an industrial laser distance measurer.^25^ The system utilizes a laser distance measurer that tracks the motion of the sternum with high precision and frequency, Fig. 1. The method is noninvasive, and causes no discomfort to the patient. Anzai (Anzai Medical, Tokyo, Japan) has recently released a resembling commercial solution but there is no published data on that solution in the literature to date.^26^

The aim of this study was to evaluate DIBH stability and reproducibility during left breast radiation treatments under the control of an in‐house developed laser‐based DIBH system for breast cancer patients. A secondary aim was to report doses to target and organs at risk (OAR).

## 2 METHODS

### 2.A Patient selection and training

Patients referred to Ålesund Hospital for left‐sided tangential radiation were eligible for the study. Twenty‐four patients requiring RT to breast only were asked for written consent to participate in the Regional Ethics Committee approved protocol. Patients had to maintain a stable breath hold for at least 20 s to be eligible for DIBH, and two patients were not able to comply with the requirement and were excluded from the analysis. The 22 patients that complied with the requirements were recruited during the period from September 2011 to August 2012; 14 patients with stage pT1‐2N0M0 left breast carcinoma and 8 patients with ductal carcinoma in situ. The patients had a median age of 58 (range 45–74) yr. Patients had no visual guidance during the first part of the training, in which the maximum breathing amplitude was found. An amplitude of 80% of maximum inhale was chosen as the DIBH‐amplitude, and a window of ±1 mm was established. Patients were then trained through audio‐visual guidance to ensure a stable breath hold during CT‐scanning. All patients were immobilized with a WingSTEP (IT‐V, Innsbruck, Austria) breast board without tilt in the supine position. Patients performed two CT‐scans, one in FB and one with DIBH. The CT scanner was a 16 slice multidetector MX8000 Brilliance IDT (Philips Medical Systems, Eindhoven, Netherlands), and images were obtained with 3 mm slice thickness. Images were transferred to Oncentra Masterplan v 3.4 (Elekta, Crawley, UK) treatment planning system.

### 2.B Treatment planning

The clinical target volume (CTV) and OARs were delineated by the same radiation oncologist inn all FB and DIBH scans. Radiation therapists delineated the lungs and external contour. The breast was delineated according to national guidelines at the time of inclusion (http://www.nbcg.no), and the heart and LAD according to other published guidelines.^27^ Planning target volume (PTV) was automatically generated, derived from CTV with 10/5/5 mm extension in the superior‐inferior/anterior‐posterior/left‐right directions (SI/AP/LR), but always 5 mm inside the external contour.

The radiation therapists made FB and DIBH treatment plans according to national guidelines and in‐house protocol. The clinical goals used in the treatment planning are listed in Table 1. 6 MV opposing tangential conformal beams with low‐weight segments were used. Wedged fields were not used in the DIBH plans to minimize the length of breath hold. Since our laser system had a fixed measuring point in the room; all patients had their isocenter placed on the sternum.

**Table 1:** Clinical goals used in the treatment planning

Structure	Goal
Heart	Max 5% of volume receive >25 Gy
Left lung	Max 15% of volume receive >20 Gy
CTV	Uniform dose 50 Gy in 25 fractions
CTV	Min 95% of volume receive 95% of 50 Gy
External	Maximum dose 55 Gy

Treatment plans were calculated with the Collapsed Cone algorithm, and originally transferred to the record and verify system Visir, but from December 2011 to Mosaiq (both Elekta, Crawley, UK) for treatment delivery.

### 2.C Treatment delivery

Treatments were delivered on either Elekta Synergy or Elekta Precise machines both equipped with 80 leaves MLC and amorphous‐silicon flat panel portal imaging systems (iView GT 3.4, Elekta, Crawley, UK). Patients had from 3 to 7 fields, and the treatment was performed within a 15 min treatment slot. The patients viewed their breathing curve through Vuzix WRAP 920 video glasses (Vuzix, West Henrietta, USA), and got instructions during the treatment session. The breathing of the patient was monitored by the radiation therapist, and the beam was manually turned on when the patient was in the correct gating window. If the patient's sternum left the gating window it was the radiation therapist's responsibility to decide if the beam should be terminated. The times at which patients entered the treatment room and the last beam was switched off were recorded.

All patients followed an offline portal imaging protocol, where the patients were imaged on day 1–3 and then weekly. The chest wall and ribs were outlined and used to match the portal image to the digitally reconstructed radiograph from the CT scan. Displacements were analyzed in the (*u,v*)‐plane for each patient (*v*‐direction parallel to CC axis and *u*‐direction perpendicular to this in AP direction). Localization offset was calculated after the 3^rd^ fraction and systematic errors were corrected. Weekly patient positioning errors of less than 5 mm were accepted; in case of having deviations over 5 mm new images were acquired and a new trend was calculated. All portal images were analyzed for systematic and random errors in accordance with the formalism proposed by van Herk et al.^28^ The average of the individual systematic setup error for the population (*μ*), the standard deviation of the individual systematic setup errors for the population (∑), and the average of the individual random setup error for the population (*σ*) was calculated.

### 2.D Statistics

Statistical analysis was made using a Wilcoxon signed‐rank test. The test was two tailed for each evaluated parameter and considered significant if *P* was <0.05. SPSS version 23 (IBM, Armonk, USA) was used in the calculations.

## 3 RESULTS

All 22 patients that complied with the requirements were able to complete their treatment sessions with DIBH. One of the two patients that were not able to comply with the requirement could not hold her breath for at least 20 s, and the other one could not comply due to psychological reasons. All patients performed a short DIBH‐training at the beginning of the first treatment session to ensure they could perform stable DIBH. The mean DIBH‐amplitude was 14 ± 4 mm. All patients complied with the standard 2 mm gating window. The median treatment session time over a treatment course was 7 min.

### 3.A Reproducibility and stability of DIBH treatment

A total of 385 portal images from 22 localized breast cancer patients treated with our in‐house DIBH technique were analyzed. The overall mean setup deviation M was smaller than 0.6 mm. The systematic error ∑, and random error *σ*, are of the same magnitude in both directions (∑*u* = 2.0 mm; ∑*v* = 1.7 mm; *σu* = 2.2 mm; *σv* = 2.1 mm), Table 2. Rotational deviations were small, not exceeding 1° overall.

**Table 2:** Overall matching results from the DIBH study with fixed laser spot

	*μu* [mm]	∑*u* [mm]	*σu* [mm]	*μv* [mm]	∑*v* [mm]	*σv* [mm]	*μ*ROT [°]	∑ROT [°]	*σ*ROT [°]
DIBH	−0.3	2.0	2.2	−0.6	1.7	2.1	−0.4	0.9	0.8

### 3.B FB versus DIBH treatment plans

Treatment planning statistics for all included patients are reported in Table 3.

**Table 3:** Comparison of average dose parameters and volumes from the FB and DIBH treatment plans

	FB	DIBH
CTV		
Dmean (Gy)	50.0 ± 0.0	50.0 ± 0.0
D_98%_ (Gy)	47.0 ± 0.5	47.0 ± 0.5
V_95%_ (%)	96.9 ± 1.1	96.9 ± 0.8
Volume (ml)	756.0 ± 400.7	759.4 ± 404.6
PTV		
Dmedian (Gy)	49.8 ± 0.1	49.8 ± 0.1
D_98%_ (Gy)	43.9 ± 3.6	45.8 ± 0.8
V_95%_ (%)	92.2 ± 2.1	92.4 ± 2.1
Volume (ml)	956.3 ± 455.5	972.1 ± 467.4
Heart		
Dmean (Gy)	3.0 ± 1.0	2.0 ± 0.9^a^
V_25 Gy_ (%)	3.3 ± 1.7	1.0 ± 1.3^a^
Volume (ml)	625.7 ± 109.7	595.5 ± 87.8
LAD		
Dmean (Gy)	28.1 ± 13.3	13.0 ± 11.4^a^
D_2%_ (Gy)	43.7 ± 11.4	29.0 ± 17.2^a^
V_20 Gy_ (%)	63.7 ± 30.0	24.4 ± 25.3^a^
Left lung		
V_20 Gy_ (%)	13.5 ± 2.5	13.3 ± 1.8
Volume (ml)	1283.1 ± 298.8	2098.2 ± 250.2^a^

a Statistically significantly (*P*<0.05) different compared with FB.

FB, free‐breathing; DIBH, deep inspiration breath hold; CTV, clinical target volume; PTV, planning target volume; LAD, left ascending coronary artery; D_98%_, dose to 98% of target volume; V_95%_, volume of target receiving 95% of prescribed dose; V_20/25 Gy_, volume of organ receiving 20/25 Gy; D_2%_, maximum dose given to 2% of volume.

### 3.C Cardiac doses

Statistically significant reduced doses were observed for the heart and LAD when using the DIBH technique as compared to FB. On average, from FB to DIBH plans, heart mean dose decreased from 3.0 ± 1.0 Gy to 2.0 ± 0.9 Gy, heart V_25 Gy_ decreased from 3.3 ± 1.7% to 1.0 ± 1.3%, LAD mean dose decreased from 28.1 ± 13.3 Gy to 13.0 ± 11.4 Gy and LAD V_20 Gy_ decreased from 63.7 ± 30.0% to 24.4 ± 25.3%. The maximum dose D_2%_ to the LAD decreased from 43.7 ± 11.4 Gy to 29.0 ± 17.2 Gy.

### 3.D Target doses

There were no significant differences between FB and DIBH plans. A total of 42 out of the 44 plans fulfilled the minimum clinical goal that at least 95% of the CTV should receive 95% of the prescribed dose. One FB and one DIBH plans did not fulfill the minimum clinical goal; 94.9% and 94.5%, respectively. The minimum dose D_98%_ to the PTV was 43.9 ± 3.6 Gy and 45.8 ± 0.8 Gy in FB and DIBH plans, respectively.

### 3.E General statistics

The mean DIBH left lung volume of the patient population was 2098.2 ml (range 1620.5–2502.1), with a 250.2 ml standard deviation; which translates to a 64% increase from the FB. There were no statistically significant differences in the mean dose to the left lung between the two techniques and the volume that received >20 Gy, V_20 Gy_, was 13.5 ± 2.5% and 13.3 ± 1.8% in the FB and DIBH groups, respectively. There were 10 plans that violated the clinical goal that only 15% of the left lung should receive >20 Gy, 5 in each group; the maximum V_20 Gy_ volume was 17.8% and 15.5% in the FB and DIBH groups, respectively. The volume of the delineated heart is significantly smaller in DIBH than in FB, 595.5 ± 87.8 ml to 625.7 ± 109.7 ml, respectively. The delineated CTVs were 756.0 ± 400.7 ml in the FB and 759.4 ± 404.6 ml in the DIBH group, and the difference was not statistically significant.

## 4 DISCUSSION

Left‐sided breast RT will to some extent irradiate the heart and increase the risk of heart disease.^29^ Darby et al. reported that the rates of major coronary events increased linearly with the mean heart dose, this increase is of 7.4% per Gy and there seems to be no lower dose threshold.^30^ The best approach would be to minimize any dose to the heart without compromising the dose to the target.^30^

**Figure 2:**
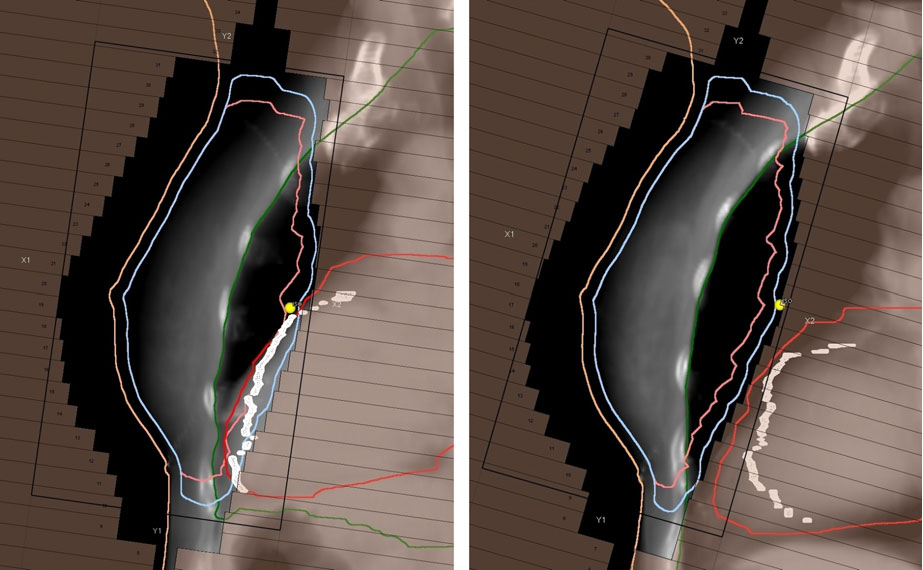
Beam's eye views of the medial tangential field in FB (left) and DIBH (right). The heart (red outline) is displaced away from the planning target volume (light blue outline) during deep inhale and the LAD (white outline) and heart are outside the treatment field.

Our clinical study assessed the interfraction setup variability with an in‐house developed DIBH system, and also evaluated the doses to OAR and target with the system. We found that the DIBH system gave a significant dose reduction in heart and LAD, while maintaining dose coverage to the clinical target, and typical beam's eye views can be seen in Fig. 2. The results are consistent with what others have presented previously.^6^, ^9^, ^10^ Anzai Medical has just released a system utilizing a distance laser measurer similar to the in‐house system we report clinical data on.^26^ Their solution will possibly be susceptible to setup variations and also varying inclination angle. Our study is the first to report clinical data on such a DIBH‐solution.

A recent review by Smyth et al. only found four studies, with a total of 69 subjects, that have reported on reproducibility or stability of DIBH treatment.^31^ Three of the studies used breast surface, and one heart position, to estimate the reproducibility and stability. There is, however, two newer studies that used 2D electronic portal image setup verification. The study by Brouwers et al. found that systematic error in voluntary breath hold patients varied between 1.4 and 1.9 mm; the random error between 2.6 and 3.3 mm.^32^ A new publication from the UK HeartSpare Study with voluntary breath hold found that the systematic error measured by electronic portal imaging in the (*u,v*)‐plane to be between 1.3 and 1.9 mm; the random error between 1.7 and 2.0 mm.^22^, ^24^ The UK HeartSpare Study found no significant difference in a randomized trail between voluntary breath hold and Elekta ABC. The systematic error in our study was −0.6 and −0.3 mm which is less than both of these voluntary breath‐hold studies, but the clinical impact of less systematic error is unknown. We have previously published a retrospective study on the reproducibility of the WingSTEP breastboard in FB, and the results from the FB patients were not significantly different from the breath‐hold population in this study.^33^ It is reported by Topolnjak et al. that portal images underestimate the actual bony anatomy setup in breast cancer patients by 20%–50% in comparison to cone beam computed tomography (CBCT), whereas Batumalai et al. did not find any difference between the two imaging techniques.^34^, ^35^

One possible reason for the small systematic error in our study might be the short treatment sessions. The median time from the patient entered the room until the last beam was turned off was 7 min in our study. The UK HeartSpare Study had a median treatment session time of 22 min.^24^ The voluntary breath‐hold technique requires no extra equipment, but the extra time to perform the technique can be costly in a busy department. In our clinic all DIBH patients are now treated during 10 min slots, and if voluntary breath‐hold patients would require 20 min slots it would greatly reduce the benefit and even require more resources. It is possible to assume that DIBH would result in better setup reproducibility as the patient for each treatment field delivered would stay in the same breath‐hold window, whereas FB patients would drift from baseline due to muscle relaxation.^36^ No studies have reported on better setup reproducibility with the DIBH technique, nevertheless our study found a moderate nonsignificant reduction in the *u*‐direction compared to retrospective in‐house data.^33^

DIBH plans provide significantly lower doses to heart and LAD than FB plans. van den Bogaard et al. recently published that left ventricle seemed to be a better predictor of acute coronary events than mean heart dose,^37^ whereas Marks et al. published that late cardiac effects were perhaps from large vessel injury.^38^ It is easy to lower the mean dose to the heart to less than 3 Gy for most patients with a DIBH technique, and it is also possible to reduce the dose to LAD if it is visualized as an OAR. The clinical importance of lowering the doses to the LAD is not yet known, and future clinical studies should address this important question.

The majority of the earlier studies that have reported on doses to organs at risk used Pencil Beam (PB) based dose calculation algorithms. Those algorithms do not take photon scatter into consideration, thereby underestimating dose outside the radiation field and potentially overestimating dose to the target when surrounded by less dense tissue. The Collapsed Cone algorithm used in our study includes photon scatter modeling, and gives a closer estimate of the dose outside the field. Vikström et al. reported on doses to organs at risk and target, and due to their use of PB algorithm, the mean doses to organs at risk in their study are lower than ours, while achieving higher minimum doses to target.^6^ Lung volume increases by 64% from FB to DIBH plans on average in our study, whereas other studies have reported on absolute lung volume increase in the range of 72%–84%.^6^, ^8^, ^9^ Our study has a lower lung volume increase, and there could be a potential for optimizing the doses even more. Vikström et al. reported the highest lung volume increase, but the study did not report on the DIBH level, and only 1 of the 17 patients went on to perform DIBH treatment.

**Figure 3:**
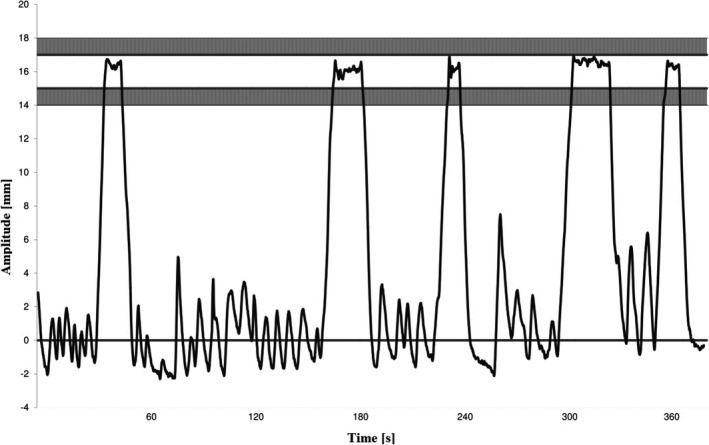
Treatment session from one patient. The gating amplitude was 16 mm with a 2 mm gating window. One test inhale before the personnel leaves the room and 4 DIBHs are performed.

A limitation of our study is that the results are based on an estimate of the dose at the time of the planning CT scan; patient contour and the inhaled volume can differ during the radiotherapy course and the setup variability is not accounted for. This can alter the dose to OARs, especially the heart and the LAD, and the coverage of the CTV could also be compromised. Some patients might also tend to flex their muscles during DIBH, something that leads to variations in how the patient returns to the baseline between two breath holds – see Fig. 3. This will again result in less inhaled air during the treatment session. There is a call for studies that take these changes into account. Another limitation of our study is the contouring of the OARs without using margins, which in particular may be relevant for heart and LAD due to heartbeats even during DIBH‐CT acquisition. White et al. showed that the minimum anisotropic margin encompassing the average 90^th^ percentile LAD motion would be 2.7 mm (LR), 4.1 mm (SI), and 2.4 mm (AP).^39^ Lorentzen et al. investigated the interobserver variation in delineating the heart and the LAD, and found that the use of guidelines reduced the spatial distance variation for heart and LAD delineations.^40^ The heart atlas by Feng et al. was used as guideline in our study.^27^ There is a decreased volume of the heart during DIBH which is probably due to increased intrathoracic pressure, and this is consistent with other studies that have found a 5%–10% reduction.^6^, ^41^ Another limitation was that the laser system had a fixed measuring point in the room; all patients had their isocenter placed on the sternum. The in‐house system has since been improved and is now capable to measure a suitable region regardless of the isocenter position.^25^

The FB plans in our study show large variation in minimum doses to the PTV. In most FB plans we had to shield the heart extensively and this also influenced the PTV coverage. A large variation in the minimum doses to the PTV would also indicate that these plans would generally not be as robust as DIBH plans; a DIBH plan would tolerate greater variations in patient setup without compromising the dose to the CTV. We found no reduction in doses to the left lung, even if the volume of the lungs increased with DIBH. The reason for this was that the increased therapeutic ratio DIBH offers was used to improve PTV coverage instead of lowering the doses to the lung. The national recommendation that maximum 5% of the heart should receive >25 Gy led to extensive shielding of the FB plans. We found that for some patients the heart follows the movement of the anterior wall of the thorax, and we could not easily improve the heart doses from FB to DIBH. All patients would benefit from DIBH, but with varying degree in regard to anatomy.

## 5 CONCLUSION

We have successfully implemented an in‐house developed DIBH system for left‐sided breast cancer patients in our clinic, and the clinical results are promising. The system was well tolerated and all patients that complied with the requirements completed their treatment sessions with DIBH. The in‐house system gave a reproducible and stable DIBH treatment verified with portal imaging. We found a significant dose reduction in heart and LAD with the DIBH system, while maintaining dose coverage to the clinical target. The most important features of our in‐house system are its simplicity, its noninvasiveness and its low cost for performing DIBH.

## CONFLICT OF INTEREST

The authors report no conflicts of interest. The authors alone are responsible for the content and writing of the paper.
